# Conserved Charged Amino Acids within Sendai Virus C Protein Play Multiple Roles in the Evasion of Innate Immune Responses

**DOI:** 10.1371/journal.pone.0010719

**Published:** 2010-05-19

**Authors:** Takashi Irie, Natsuko Nagata, Tomoki Igarashi, Isao Okamoto, Takemasa Sakaguchi

**Affiliations:** Department of Virology, Graduate School of Biomedical Sciences, Hiroshima University, Hiroshima, Japan; Fundação Oswaldo Cruz, Brazil

## Abstract

One of the accessory proteins of Sendai virus (SeV), C, translated from an alternate reading frame of P/V mRNA has been shown to function at multiple stages of infection in cell cultures as well as in mice. C protein has been reported to counteract signal transduction by interferon (IFN), inhibit apoptosis induced by the infection, enhance the efficiency of budding of viral particles, and regulate the polarity of viral genome-length RNA synthesis to maximize production of infectious particles. In this study, we have generated a series of SeV recombinants containing substitutions of highly conserved, charged residues within the C protein, and characterized them together with previously-reported C′/C(−), 4C(−), and F170S recombinant viruses in infected cell cultures in terms of viral replication, cytopathogenicity, and antagonizing effects on host innate immunity. Unexpectedly, the amino acid substitutions had no or minimal effect on viral growth and viral RNA synthesis. However, all the substitutions of charged amino acids resulted in the loss of a counteracting effect against the establishment of an IFN-α-mediated anti-viral state. Infection by the virus (Cm2′) containing mutations at K77 and D80 induced significant IFN-β production, severe cytopathic effects, and detectable amounts of viral dsRNA production. In addition to the Cm2′ virus, the virus containing mutations at E114 and E115 did not inhibit the poly(I:C)-triggered translocation of cellular IRF-3 to the nucleus. These results suggest that the C protein play important roles in viral escape from induction of IFN-β and cell death triggered by infection by means of counteracting the pathway leading to activation of IRF-3 as well as of minimizing viral dsRNA production.

## Introduction

Sendai virus (SeV; mouse parainfluenza virus type 1), a prototype of the family *Paramyxoviridae* of the order *Mononegavirales* which includes some of the most important and ubiquitous disease-causing viruses of humans and animals, such as measles virus, parainfluenza viruses, mumps virus, Nipah virus, Hendra virus, human metapneumovirus, Newcastle disease virus, canine distemper virus, and rinderpest virus, contains a nonsegmented, negative-stranded RNA genome with length of 15,384 nucleotides [1]. This genome encodes six viral structural proteins, a nucleprotein (N), a highly phosphorylated component of the viral RNA-dependent RNA polymerase (vRdRp) complex (P), a matrix protein (M), a glycoprotein with haemagglutinin-neuraminidase activity (HN), a glycoprotein with membrane-fusion activity (F), and a large catalytic subunit of the vRdRp complex (L), tandemly in this order [1]. Both the replication of genome-length RNAs, including negative (−)-sense genome and positive (+)-sense antigenome RNAs, and the transcription of viral messenger RNAs (mRNAs) are carried out by a vRdRp mainly composed of the L and P proteins [1].

The Paramyxovirus P gene is unique in producing more than one polypeptide species. The SeV P gene is the most diverse of the paramyxovirus P genes, with at least seven polypeptides expressed from it: in addition to P protein, four C proteins (C′, C, Y1, and Y2) are translated from start codons in the +1 reading frame relative to the P open reading frame (ORF), and proteins V and W are produced from the altered P ORF with the insertion of one or two G residues at a specific position of the mRNA, respectively, during transcription [1].

The SeV C proteins have been shown to have multiple functions during viral replication in cell cultures (*in vitro*) and in mice (*in vivo*). The best-characterized of these functions is interruption of the Jak/STAT signaling pathway after stimulation by type I IFNs, resulting in a lack of activation of IFN-stimulated genes (ISGs) and establishment of an antiviral state in the infected cells [2], [3], [4], [5], [6]. C proteins have been shown to physically interact with cellular signal transducers and activator of transcription (STAT) 1, and to inhibit tyrosine phosphorylation of STAT1 and STAT2 and dephosphorylation of phosphorylated STAT1 by an unidentified mechanism [4], [7], [8], [9]. The C terminal half of C protein is sufficient for this function, since the four C proteins of SeV are virtually indistinguishable with regard to their antagonizing effects on responses to IFN [6], and deletion of the N-terminal half does not abolish this ability, though shortening 14 amino acids of the C-terminal does [5]. Recently, it has been reported that the substitution of three charged amino acid residues at positions 151, 153, and 154 within C protein conserved among SeV strains dramatically reduced the anti-IFN ability without any alteration of the other functions of C protein in experiments with C protein expressed from cDNA as well as infections of SeV recombinants [10], [11]. These substitutions led to an attenuation of the SeV recombinant in mice in a STAT1-dependent manner, but not to reduced viral growth in cell cultures [10].

SeV C protein is also reported to inhibit apoptosis induced by the infection through an undefined mechanism. The apoptosis induced by VSV was efficiently inhibited by pre-infection with wild-type (WT) SeV but not a SeV recombinant lacking C protein expression [12]. Infections of SeV recombinants lacking the expression of C protein or containing an F170S mutation within the protein leads to a dramatically quicker and severer cytopathology due to apoptosis than infections of SeV-WT [3], [12].

In addition, C protein has been reported to regulate viral RNA synthesis. Earlier studies reported that the elimination of C protein expression from the P gene increased mRNA synthesis in infected cells and supplementation with C protein from cDNA eliminated this increase [13], [14], and that *trans*-supplied C proteins inhibited viral replication [15]. Detailed studies using mini replicons mimicking SeV defective-interfering (DI) genomes have shown that C protein inhibited synthesis of positive-sense viral RNAs including viral mRNAs and antigenomic RNA from the leader promoter at the extreme 3′-end of viral genomic RNA [13], [16]. Such inhibitory effects of C protein on viral RNA synthesis have been suggested to be exerted through physical interaction between C and L proteins, since the strength of C-L interaction correlated with defects in viral RNA synthesis in experiments using a series of C mutants containing substitutions of highly conserved, charged amino acids [11], [17]. We have recently reported that SeV C protein regulates viral RNA synthesis to optimize production of infectious particles probably by controlled inhibition of positive-sense viral RNA synthesis in the course of viral replication [18]. Production of non-infectious viral particles containing (+)-sense antigenomic RNA was markedly increased compared to that of (−)-sense genomic RNA-containing infectious particles in cell cultures infected with C-deficient SeV recombinants [18].

SeV C proteins function also in the formation and budding of viral particles. Lack of C protein expression has been reported to reduce production of infectious particles and increase production of heterogeneous particles [18], [19]. In addition, C protein has been shown to enhance the budding of virus-like particles (VLPs) formed by SeV M protein by recruiting Alix/AIP1, a cellular protein involved in apoptosis and endosomal trafficking, to the plasma membrane through physical interaction [20], [21].

In this paper, a series of SeV recombinants containing substitutions of highly conserved, charged amino acid residues within C proteins were characterized exhaustively in terms of growth kinetics, viral protein synthesis, release of viral particles, genomic and antigenomic RNA replication, ability to inhibit induction of an antiviral state triggered by IFN treatment, inducibility of IFN-β in the infected cells, and inhibitory effect on poly(I:C)-mediated activation of cellular IRF-3. It was found that the charged amino acids conserved within C protein play multiple roles in the evasion of host innate immunity, by means not only of the well-characterized ability to antagonize IFN signaling but also of minimizing viral dsRNA production and counteracting the pathway leading to IRF-3 activation.

## Results

### Recovery and growth kinetics of a series of SeV recombinants with mutations in their C proteins

**Figure 1:**
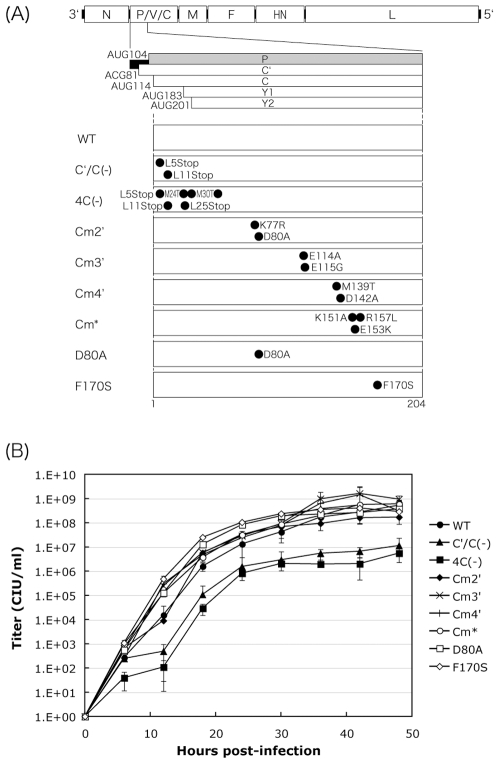
SeV recombinants used and their growth kinetics. (A) Schematic representation of the SeV genome highlighting the start region of the P gene including the C ORF. The amino acid changes for the various mutants are indicated. (B) Graphs of one-step growth kinetics of the viruses in LLC-MK_2_ cells. Each titer represents the average for at least three independent experiments.

It has been reported that clusters of charged amino acids conserved within C proteins among SeV strains are important for the inhibition of (+)-sense viral RNA synthesis [17], and that some of the charged amino acids play an important role in the inhibitory action against IFN signaling [11]. To examine the importance of the charged amino acids to the functions of C protein in the course of an infection, we generated a series of SeV recombinants, Cm2′, Cm3′, Cm4′, and D80A, containing changes to clustered, charged amino acids within C proteins with reference to a previous report [17] (Fig. 1A). The substitutions introduced into these viruses were not exactly the same as those in the previous report where all of the charged amino acids were replaced by alanines [17]. The substitutions were made so as not to alter the P polypeptide in the overlapped, shifted C ORF (Fig. 1A). A recombinant SeV, F170S, possessing an F170S mutation within the C protein was also generated. The F170S mutation has been reported to lead to an attenuation of virulence in mice, loss of the ability to antagonize IFN signaling, and increased induction of apoptosis without affecting virus growth in cell cultures [3], [4], [22]. The previously reported SeV recombinants, C′/C(−) and 4C(−), in which the expression of C′ and C, and all four C proteins is knocked out, respectively [23], and the Cm* recombinant containing triple amino acid substitutions, K151A, E153K, and R157L, in the C protein resulting in a loss of anti-IFN capacity without a change in virus multiplication in cell cultures [10] were also used in this study.

**Figure 2:**
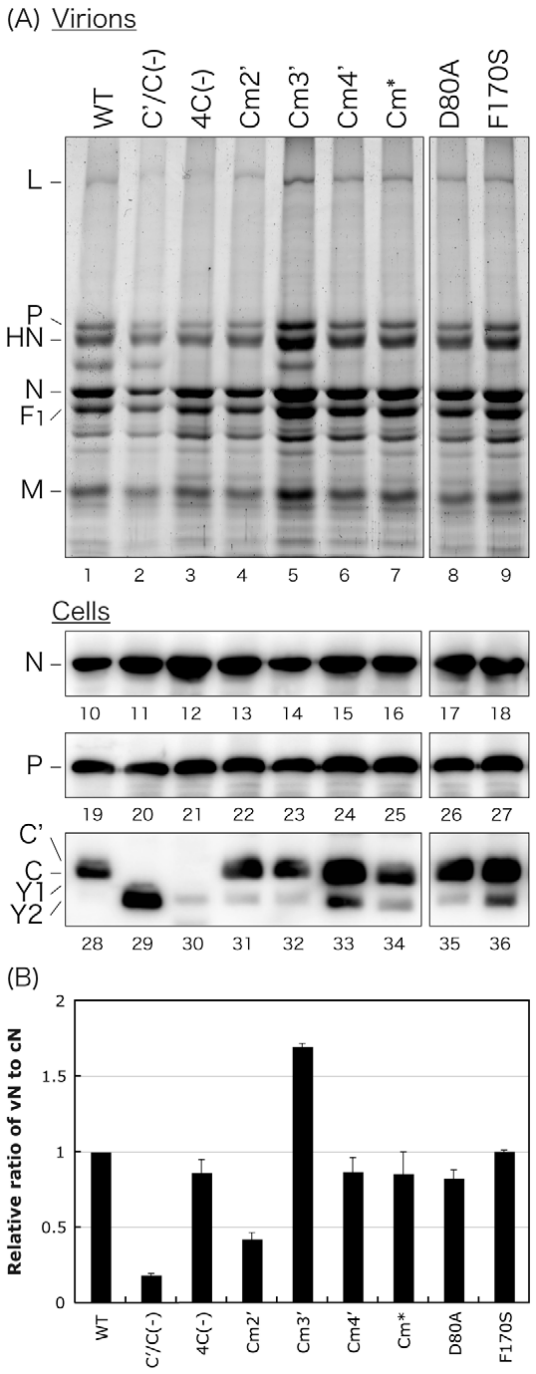
Protein profiles for WT and recombinant viruses. (A) SDS-PAGE analysis of virion proteins released and accumulated for 48 h p.i. in the culture medium of LLC-MK_2_ cells infected with the indicated viruses and Western blots for N, P, and C protein expression in the infected cells. (B) The amounts of N protein in virions (vN) and in the infected cells (cN) in Fig. 2A were quantitated and the ratio of vN to cN is shown as a bar graph. The ratio in SeV-WT was set to 1. Bars represent the average for three independent experiments.

We first examined the growth kinetics of these C mutant viruses with a one-step growth curve in LLC-MK_2_ cells (Fig. 1B). As reported [23], overall titers of the C′/C(−) and 4C(−) viruses were reduced by 1 to 2 logs compared to those of SeV-WT (Fig. 1B). In contrast, titers of the other C mutant viruses were not markedly different than those of SeV-WT. Titers of Cm2′ and Cm3′ were the lowest and highest, respectively, among the viruses tested during the course of the experiments (Fig. 2B).

### Profiles of viral protein expression

Since we and others have previously reported that lack of expression of C proteins affected the synthesis of viral proteins and the budding efficiency of viral particles [18], [23], production of viral proteins in the cells infected with the C mutant viruses and release of virion proteins into the culture medium were examined by SDS-PAGE and Western blotting using viral protein-specific antibodies (Fig. 2). Consistent with the previous report [18], viral N protein expression was slightly increased in the 4C(−)-infected cells compared to that in the SeV-WT-infected cells (Fig. 2, lanes 10 and 12). The expression of viral N protein in the cells infected with the other C mutant viruses was not markedly different from that in the WT-infected cells, although the amount of N protein in the cells infected with the Cm4′ and F170S viruses was slightly increased (Fig. 2A, lanes 10–18). Similar results were observed for the expression of P protein in the infected cells, confirming that the mutations introduced into the C ORF did not affect the protein synthesis from the P ORF (Fig. 2A, lanes 19–27). As for C proteins, the expression of C protein was dominant over than that of C′, Y1, and Y2 proteins and a much smaller amount of Y2 and a trace amount of Y1 proteins were detected in the cells infected with the viruses tested except for the C′/C(−) and 4C(−) viruses (Fig. 2A, lanes 28–36). In the 4C(−)-infected cells, elimination of the expression of all four C proteins was confirmed (Fig. 2A, lane 30), and consistent with the report by Kurotani *et al.* [23], C′ and C protein expression was also knocked out in the C′/C(−)-infected cells, but the expression of Y2 protein was increased compared to that observed in the WT virus-infected cells (Fig. 2A, lane 29).

The protein profiles of the virions purified by ultracentrifugation through a sucrose cushion from the culture medium of the cells infected with the indicated viruses were also compared by SDS-PAGE (Fig. 2A, lanes 1–9). The composition of virion proteins of each mutant virus was virtually identical to that of the WT virion. As reported [18], the amount of virion protein of C′/C(−) detected in the culture medium was reduced, and that of 4C(−) was virtually unchanged compared to that of SeV-WT (Fig. 2A, lanes 1–3). As for the other viruses except for Cm2′ and Cm3′, the amount of virion protein was virtually identical to that of the WT (Fig. 2A, lanes 1 and 4–10). Consistent with the slightly reduced viral titers and the highest titers of the viruses tested, the amounts of Cm2′ and Cm3′ virion proteins were slightly lower and higher than the amount of the WT virion protein, respectively (Fig. 2A, lanes 4 and 5).

The N protein bands of virion samples (vN) and cells (cN) were quantified, and the ratios of vN to cN were compared to examine the efficiency of viral release (Fig. 2B). As reported [18], the vN/cN ratios of the C′/C(−) and 4C(−) viruses were reduced almost 90% and only 10–20%, respectively, despite an almost 2-log reduction in infectivity compared to that of the WT virus (Fig. 2B). As for the other C mutant viruses, except for Cm2′ and Cm3′, the ratios were virtually identical to that of SeV-WT. As expected from their viral infectivity observed in their one-step growth kinetics, the ratios of the Cm2′ and Cm3′ viruses were 60% lower and 70% higher than that of the WT virus, respectively (Fig. 2B).

### Polarity of viral genomes in particles

**Figure 3:**
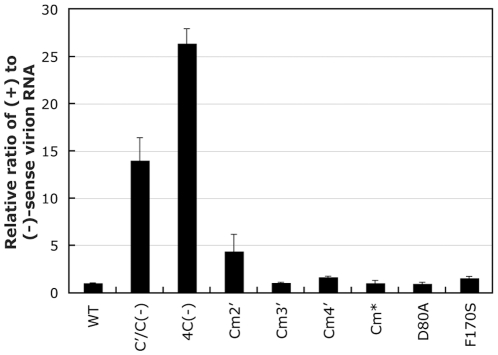
Viral genomic and antigenome-sense RNA incorporated in virions. Ratios of (+)- to (−)-sense RNAs in the indicated virions, which were detected by two-step qRT-PCR, are shown as a bar graph. The ratio of the WT-sample was set to 1. Bars represent the average for three independent experiments.

It has been reported that C proteins inhibit the synthesis of (+)-sense viral RNA including antigenomic RNA and viral mRNA probably through direct interaction with viral L protein [17]. In addition, we have recently reported that C proteins regulate (+)-sense viral RNA synthesis to maximize production of infectious particles containing (−)-sense viral genomic RNA but not that of uninfectious particles containing (+)-sense antigenomic RNA [18]. To examine whether the mutations introduced into the charged amino acids within the C proteins would affect efficiency of production of (−)-sense genomic RNA-containing infectious particles, we next compared the composition of (−) and (+)-sense RNAs in the virions released from the cells infected with the C mutant viruses (Fig. 3). As we recently reported [18], the ratio of (+) to (−)-sense RNA in the C′/C(−) and 4C(−) virions released from the infected cells into the culture medium was remarkably higher (approximately 14 and 26-fold, respectively) than that observed in the WT virions, indicating that the particles produced from the infected cells include (+)-sense antigenomic RNA-containing non-infectious virions at a ratio much higher than that observed in the WT virions (Fig. 3). As for the other C mutant viruses, the (+)/(−) ratio in virions was virtually identical to that detected in the WT virion except in the Cm2′ virion, in which it was 4.5-fold higher than that in the WT virion (Fig. 3). These results indicate that the conserved charged amino acids in C proteins do not play critical roles in the regulation of viral RNA synthesis at the level observed in the C-knock out viruses.

The mutations in the conserved, charged amino acids are not suggested to dramatically affect the efficiency of viral replication in cell cultures, although a slight reduction in the release of particles and increase in production of (+)-sense antigenomic RNA-containing particles were observed for the Cm2′ virus.

### Cytopathic effect of the C mutant viruses

**Figure 4:**
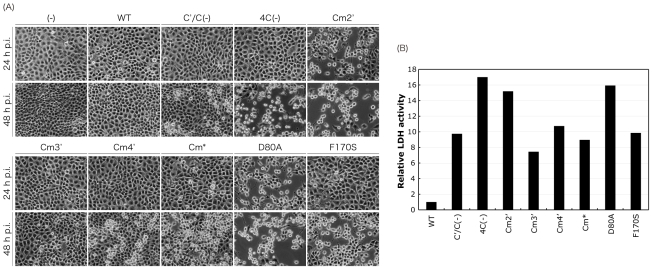
Cytopathogenicity of the WT and recombinant viruses. (A) Microscopic analysis of virus-induced CPE. LLC-MK_2_ cells were mock-infected or infected with the indicated viruses at an MOI of 5 and cells were observed at 24 and 48 h p.i. (B) Cytotoxicity assay using LLC-MK_2_ cells. LLC-MK_2_ cells were infected with the indicated viruses at an MOI of 5. The lactate dehydrogenase (LDH) activity released from the damaged cells for 48 h after infection was measured as described in the Materials and Methods section. The value of the WT-sample was set to 1.

C proteins have also been reported to inhibit apoptosis induced by infection [12]. In fact, as reported [12], the viruses lacking C protein expression, C′/C(−) and 4C(−), had a mild and much greater cytopathic effect (CPE) at a later time point after infection (48 h p.i.) in LLC-MK_2_ cells, although SeV-WT showed virtually no CPE at this time point (Fig. 4A). CPEs induced by infections of the other C mutant viruses were also observed at earlier (24 h p.i.) and later (48 h p.i.) time points in LLC-MK_2_ cells (Fig. 4A). The Cm3′ virus showed little CPE similar to the WT virus throughout the experiment, and the CPE observed in the cells infected with Cm4′ and Cm* was slightly severer than that observed in the WT-infected cells at the later time point. As expected from previous reports [3], [22], the F170S virus showed a severer CPE than the WT as well as Cm3′ and Cm* viruses, although it was slightly milder than that of the 4C(−) virus. Surprisingly, the Cm2′ virus showed a much quicker and severer CPE than the other viruses including even the 4C(−) virus. Indeed, Cm2′ showed a severe CPE at 24 h p.i., comparable to that of the 4C(−) virus observed at 48 h p.i., and at 48 h p.i., almost all cells were rounded or detached. A similar result was obtained for the D80A virus possessing one of the amino acid substitutions introduced into the Cm2′ virus, indicating that only the D80A mutation of the C protein was responsible for the phenotype. The viability of the cells infected with the C mutant viruses almost paralleled the results in Fig. 4A (Fig. 4B), confirming that the CPEs observed in the cells infected with the C mutant viruses were the result of cell killing induced by the infection. These results indicate that some of the conserved, charged amino acids such as K77 and D80 play critical roles in the cell-killing ability of SeV, or conversely, in the ability to inhibit cell-killing induced by SeV infection.

### Ability of the C mutant viruses to antagonize IFN signaling

**Figure 5:**
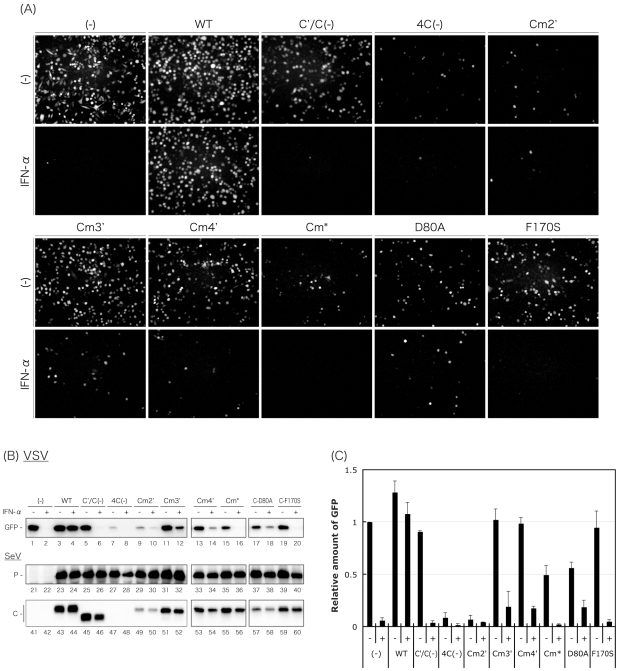
Ability of the WT and recombinant viruses to rescue VSV from the anti-viral action of IFN-α. HeLa cells were mock-infected or infected with the indicated viruses. At 6 h p.i., the media were replaced with fresh media containing IFN-α (1,000 IU/ml) or no IFN-α. After an additional 6-h incubation, cells were superinfected with rVSV-GFP. After further incubation for 6 h, GFP expression derived from rVSV-GFP replication was observed under a fluorescent microscope. (B) Cell lysates were analyzed by Western blotting to detect expression of rVSV-GFP-derived GFP, SeV P, and C proteins. (C) The amounts of GFP in the cell lysates of Fig. 5B were quantitated and shown as a bar graph. The value of the mock-infected, IFN-α-non-treated sample was set to 1. Bars represent the average for three independent experiments.

SeV C proteins have been well demonstrated to antagonize IFN signaling [2], [3], [4], [5], [6], [7], [8], [9], and the charged amino acids introduced into the Cm* virus were reported to be important for this function [10], [11]. We next examined the effect of the C mutant viruses on the induction of an antiviral state triggered by IFN-α treatment (Fig. 5). Briefly, HeLa cells infected with the SeV recombinants were treated with IFN-α, and then superinfected with rVSV-GFP. The production of GFP by replication of rVSV-GFP in the cells was examined by fluorescent microscopy and Western blotting using an anti-GFP antibody (Fig. 5).

In the microscopic analysis (Fig. 5A), GFP-fluorescence was not detected after IFN-α treatment in uninfected cells due to the induction of an antiviral state by the treatment, while almost all cells not treated with IFN-α showed GFP-fluorescence as expected. In contrast, as reported [24], numbers of GFP-fluorescent cells were virtually unchanged in the SeV-WT-infected cells, regardless of IFN-α treatment, due to the inhibitory effect of the SeV-WT infection against an IFN-α-triggered antiviral state. In contrast, the results obtained with the cells infected with the C′/C(−), Cm3′, Cm4′, and F170S viruses were similar to those for the uninfected cells, indicating that these viruses have lost the ability to inhibit the induction of an antiviral state by IFN-α treatment. Interestingly, among the cells infected with the 4C(−), Cm2′, Cm*, and D80A viruses, numbers of GFP-fluorescent cells were also reduced dramatically by IFN-α treatment, but reduced remarkably even in the absence of IFN-α, suggesting that the infection itself was able to induce an antiviral state without IFN treatment, and that the charged amino acids substituted within the Cm2′, Cm*, and D80A viruses played important roles in this function.

GFP expression was also monitored by Western blotting using anti-GFP antibody (Fig. 5B, lanes 1–20), and the protein bands of GFP were measured and represented as a bar graph (Fig. 5C) to confirm the observation of Fig. 5A. Expression of P protein was virtually identical between samples infected with the recombinant viruses, confirming their almost equal levels of replication (Fig. 5B, lanes 21–40). Expression of C proteins was also examined by Western blotting using anti-C pAb, and levels of C protein expression did not differ greatly between the samples except for those samples infected with the Cm2′ and D80A viruses where levels of C proteins were reduced despite an equivalent expression of P protein to that in SeV-WT-infected cells.

### Induction of IFN-β production in the cells infected with the C mutant viruses

**Figure 6:**
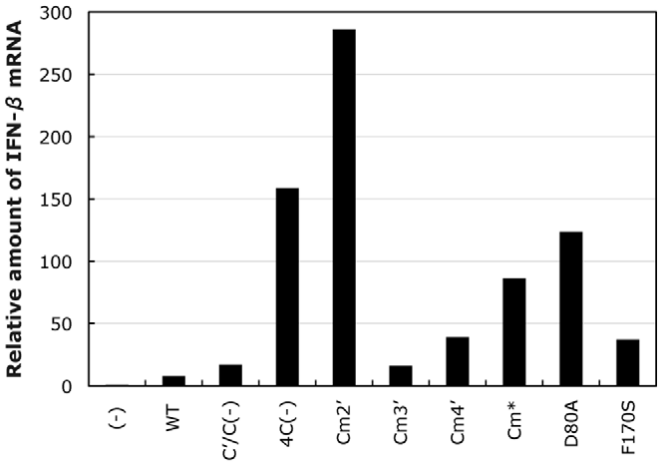
IFN-β production in the HeLa cells mock-infected and infected with the indicated viruses. The relative amounts of IFN-β mRNA detected in the cells infected with the indicated viruses at 48 h p.i. revealed by one-step qRT-PCR are shown as a bar graph. The amount of the mRNA detected in the mock-infected sample was set to 1.

The antiviral state induced by the Cm2′, Cm*, and D80A viruses without IFN treatment might be due to the production of IFNs in the infected cells. To assess this possibility, levels of IFN-β mRNA in the cells infected with the C mutant viruses were compared using a quantitative RT-PCR (Fig. 6). As expected, amounts of IFN-β mRNA were much higher in the cells infected with 4C(−), Cm2′, Cm*, and D80A (approximately 160, 290, 90, and 130-fold, respectively) than in the uninfected cells, while levels in the SeV-WT-infected cells were increased only 6-fold. In the cells infected with the other viruses, expression of IFN-β mRNA was also increased compared to that in the WT-infected cells (ranging from 2.2 to 5.7-fold), but the levels were lower than those observed in the cells infected with the IFN-β-inducing recombinant viruses. Together with the results in Fig. 5, this indicates that the mutations in some of the C mutant viruses affect the ability of the viruses to induce IFN-β expression as well as to inhibit IFN-evoked responses in the infected cells, and strongly suggests that that charged amino acids, K77, D80, K151, E153, and R157, play important roles in the ability of the C proteins to induce IFN-β expression by SeV infection.

### Synthesis of dsRNA in the cells infected with the C mutant viruses

**Figure 7:**
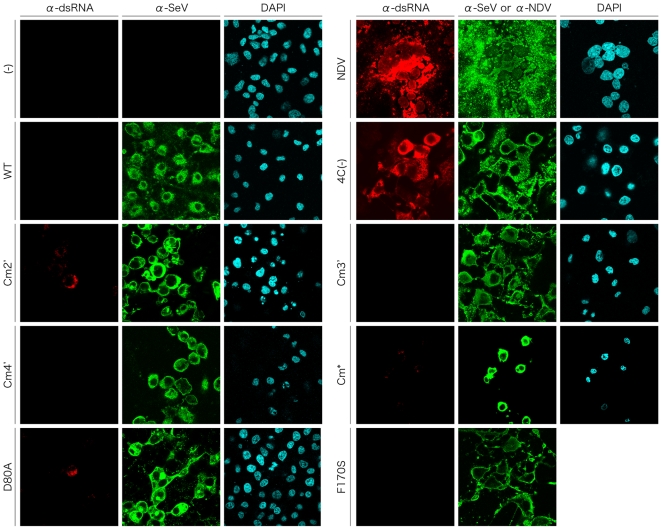
Immunofluorescent staining of the SeV-infected cells with an anti-dsRNA mAb. HeLa cells were mock-infected or infected with the indicated viruses. At 36 h p.i., cells were fixed, permeabilized, and then stained with an anti-dsRNA mAb J2, anti-SeV pAb, and DAPI as described in the Materials and Methods section. Cells were observed under a Zeiss LSM5 confocal microscope.

It has been believed that RNA species such as dsRNA and 5′-triphosphate RNA, produced in cells infected with an RNA virus, are sensed by cellular RNA sensors, such as retinoic acid-inducible gene I (RIG-I) and melanoma differentiation associated gene 5 (MDA5), leading to production of cytokines including IFN-β [25], [26], [27], [28], [29]. Recently, dsRNA was reported to be detected in cells infected with recombinant SeVs lacking the expression of C proteins, but not in WT virus-infected cells [30]. To address the possibility that the induction of IFN-β expression by SeV recombinants is triggered by the production of dsRNA in the infected cells, we next examined dsRNA production in the cells infected with the C mutant viruses by immunofluorescent staining using an anti-dsRNA antibody (Fig. 7). As reported [30], fluorescent signal of dsRNA was readily detectable in the cells infected with NDV, while it was not detected in uninfected cells. As for SeV, consistent with the previous report, dsRNA was not detected in the cells infected with the WT virus, as in the uninfected cells, but was clearly observed in the 4C(−)-infected cells. The fluorescent signals of dsRNA in the cells infected with NDV and 4C(−) was eliminated by treatment of the fixed, infected cells with RNase III which specifically cleaves dsRNA, before staining with the anti-dsRNA antibody, confirming that the fluorescent signals were specific to dsRNA (data not shown). In the cells infected with most of the other mutant viruses except Cm2′, Cm*, and D80A, no fluorescence was detected. However, in the Cm2′- and D80A-infected cells, fluorescent-positive cells were detected at a much lower frequency than that observed in the 4C(−)-infected cells. Fluorescence of dsRNA was hardly detectable, but weak signal was observed in the Cm*-infected cells, which was stronger than in the WT-infected cells. These four-dsRNA producing viruses, 4C(−), Cm2′, Cm*, and D80A, produced the highest IFN-β levels in the infected cells (compare Figs. 6 and 7), and levels of dsRNA detected in the infected cells were roughly correlated with levels of IFN-β production induced by infection, implying a link between the ability of the C mutant viruses to induce IFN-β production and to produce dsRNA in the infected cells.

### Inhibition of poly(I:C)-induced translocation of IRF-3 into the nucleus by infection of the C mutant viruses

**Figure 8:**
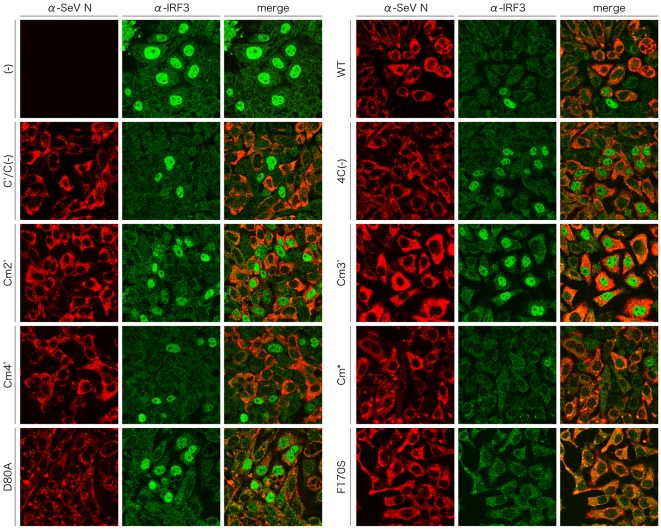
Subcellular distribution of cellular IRF-3 in the SeV-infected cells after induction by poly(I:C). HeLa cells were mock-infected or infected with the indicated viruses. At 12 h p.i., cells were transfected with 5 µg of poly(I:C) and incubated for another 6 h. Cells were fixed, permeabilized, and then stained with anti-IRF-3 pAb and anti-SeV N mAb as primary antibodies. Cells were observed under a Zeiss LSM5 confocal microscope.

Finally, we examined whether the C mutant viruses could inhibit the innate immune responses triggered by the introduction of poly(I:C) into infected cells. For this purpose, the subcellular distribution of cellular IRF-3 was observed after poly(I:C) treatment of the cells infected with the C mutant viruses (Fig. 8). Cellular IRF-3 was predominantly detected in the nucleus after poly(I:C) treatment of uninfected cells (Fig. 8), while diffusely-distributed in the cytoplasm without the treatment (data not shown). In the cells infected with the WT, C′/C(−), Cm4′, Cm*, and F170S viruses, nuclear translocation of IRF-3 induced by poly(I:C) treatment was detected in the SeV antigen-negative but not positive cells, indicating that the poly(I:C)-induced translocation of IRF-3 to the nucleus was inhibited by these viruses. In contrast, in the cells infected with the 4C(−), Cm2′, Cm3′, and D80A viruses, the nuclear localization of IRF-3 after poly(I:C) treatment was observed even in SeV-antigen-positive cells. These results indicate that C proteins have the ability to inhibit the pathway from the recognition of poly(I:C) by the cellular RNA sensors to the activation and translocation of IRF-3, and suggest that some of the charged amino acids, especially K77, D80, E114, and E115, play an important role in this function.

## Discussion

The SeV C proteins have been shown to exert multiple actions at multiple steps of infection *in vitro* and *in vivo*; making it difficult to evaluate each function individually in the course of an infection. For example, the C′/C(−) and 4C(−) viruses, which lack expression of the C protein partly and totally, respectively, showed extensive deficiencies in replication in the cell cultures as well as in mice. This is thought to be due to the combined effects of aberrant formation and budding of viral particles, uncontrolled synthesis of viral RNAs, and loss of antagonizing ability against IFN responses and inhibitory effect against the induction of apoptosis [2], [12], [18], [19], [23], [24]. Previous studies using a spontaneous SeV mutant, Ohita-MVC11, isolated from the virulent Ohita-M1 strain have indicated that even a single amino acid substitution of F170S within the C proteins, which led to severe attenuation in mice, modified more than one function associated with the C proteins [3], [22]. In experiments using recombinant C proteins, C-terminal deletion of the C proteins resulted at least in the loss of ability to antagonize IFN responses as well as that to enhance SeV M-VLP budding [5], [20], [31]. In addition, a series of substitutions of highly conserved, charged amino acid residues within the C protein altered the ability to inhibit viral RNA synthesis from the leader promoter in a mini replicon system and to counteract IFN responses [11], [17]. Here, we generated a series of SeV recombinants containing single to triple substitutions of conserved, charged amino acid residues within the C protein without affecting the P ORF and analyzed their infection characteristics in cell cultures compared with the previously characterized SeV recombinants, C′/C(−), 4C(−), and F170S. We found that the C protein antagonized host innate immunity with multiple strategies, not only by its counteraction of IFN responses through a Jak/STAT pathway (summarized in Table 1).

**Table 1:** Properties of the recombinant viruses used in this study.

Virus	CPEa)	Inhibition of IFN-α-triggered antiviral stateb)	Induction of antiviral statec)	Induction of IFN-βd)	Generation of dsRNAe)	Inhibition of poly(I:C)-mediated translocation of IRF-3g)
WT	−	+	−	−	−	+
C′/C(−)	+	−	−	−	n.d.f)	+
4C(−)	++	−	++	++	+++	−
Cm2′	+++	−	++	++	++	−
Cm3′	−	−	−	−	−	−
Cm4′	+	−	−	−	−	+
Cm*	+	−	+	+	+	+
D80A	+++	−	+	+	++	−
F170S	++	−	−	−	−	+

a) CPE of the recombinants shown in Fig. 4A (−, little or no; +, slight; ++, severe; +++, quite severe).

b) Inhibitory effect of the recombinants against IFN-α-induced antiviral state examined in Fig. 5.

c) Inducibility of antiviral state by infection of the recombinants examined in Fig. 5 (−, no; +, moderate; ++, high).

d) Levels of IFN-β induced by infection of the recombinants examined in Fig. 6 (−, 1–50-fold; +, 50–150-fold; ++, >150-fold higher than that of uninfected sample, (−)).

e) dsRNA synthesis in the cells infected with the recombinants examined in Fig. 7 (−, no; +, slight; ++, weak; +++, strong).

f) n.d., not done.

g) Inhibitory effect of the recombinants against poly(I:C)-mediated translocation of IRF-3 into the nucleus examined in Fig. 8.

Unexpectedly, none of the mutations within the C proteins introduced in this study dramatically affected viral replication in LLC-MK_2_ cells. Indeed, growth kinetics, the release of viral particles from the infected cells into the culture medium, viral protein synthesis in the infected cells, and ratios of particles containing (+)- and (−)-sense genome-length RNAs were not significantly different between the WT and C recombinant viruses except for the C-deficient viruses, C′/C(−) and 4C(−), though only the Cm2′ virus showed a relatively small impairment of replication, with a slight reduction in titer and release of particles, and increase of production of (+)-sense antigenome RNA-containing non-infectious virions compared to the WT virus.

In previous studies using cDNA, alanine substitutions of conserved, charged amino acid residues have been shown to reduce the ability of the C protein to inhibit viral RNA synthesis from the leader promoter in a mini replicon system [11], [17]. Although such alterations of C protein function would be expected to result in an increase in the production of (+)-sense antigenome RNA-containing non-infectious particles, no significant difference was observed in infectivity between the WT and the mutant viruses. This discrepancy may be due to the difference in the amino acids that were substituted; in the previous reports [11], [17], all amino acids were substituted with alanines, but in this paper, substitutions were highly limited due to the restriction of not affecting the P ORF. Alternatively, it may be due to the difference in experimental systems; a simplified protein expression system from cDNA in the previous reports, and a complicated system using live recombinant viruses in this report.

As for the antagonizing effect of C proteins against IFN responses through a Jak/STAT pathway, infection of all of the SeV recombinants, Cm2′, Cm3′, Cm4′, and Cm*, containing substitutions of the charged amino acid residues had lost their counteraction against induction of an antiviral state triggered by IFN-α treatment, suggesting that K77, D80, E114, E115, D142, K151, E153, and R157 within the C proteins are all important for this function. Again, there was a discrepancy between this result and those of previous reports in that the amino acid substitutions K151A, E153A, and R157A but not K77A and D80A, E114A and E115A, and M139A and D142A diminished the antagonizing capability, probably due to the difference in the amino acids substituted and/or in the experimental system used, as mentioned above.

More interestingly, an antiviral state was fully induced by the infection of Cm2′ itself at a similar level to the case of 4C(−) infection, and partially by the Cm* and D80A viruses. The induction was almost correlated with IFN-β production in the infected cells. This observation strongly suggests the importance of the C proteins in viral circumvention of host innate immunity as typified by IFN-β production.

The infection of host cells has been known to be sensed by cellular RNA helicases, RIG-I and MDA5, which have differential roles in the recognition of different viruses [27]. RIG-I has been to be believed to detect many RNA viruses including paramyxoviruses, orthomyxoviruses, rhabdoviruses, and filoviruses probably by recognizing 5′-triphosphated RNA and short dsRNA produced during viral RNA synthesis [25], [26], [29], while MDA5 has been reported to detect picornaviruses by recognizing long dsRNA [26], [27], [28]. In fact, dsRNA was observed in the cells infected with the IFN-inducible viruses, 4C(−), Cm2′, Cm*, and D80A, to varying degrees. SeV C proteins have been shown to regulate viral RNA synthesis from the leader promoter including production of viral antigenomic RNA and mRNAs [13], [16]. Viral leader RNA synthesized during transcription of viral mRNAs is a 5′-triphospated RNA, and believed to be recognized by RIG-I, followed by induction of IFN-β [32]. Loss of the ability of the C protein to regulate viral RNA synthesis might increase leader RNA production, resulting in increased induction of IFN-β. In addition, lack of C protein expression has been reported to increase aberrant positive-sense viral RNA production at least containing the region spanning from the leader to the M gene [18]. Such aberrant RNA might serve as a trigger to induce host innate immune responses. SeV C proteins might regulate viral RNA synthesis to optimize production of (−)-sense viral genomic RNA-containing infectious particles as well as to minimize synthesis of viral RNA which is recognized by RIG-I and/or MDA5 and triggers host innate immunity.

SeV C proteins also seem to have the ability to inhibit a signaling pathway from the recognition of dsRNA by MDA5 to activation of IRF-3. Translocation of IRF-3 to the nucleus triggered by a synthetic dsRNA, poly(I:C), which is know to be recognized by MDA5, was inhibited in the cells infected with the WT, C′/C(−), Cm4′, Cm*, and F170S viruses, but not in those infected with the 4C(−), Cm2′, Cm3′, and D80A viruses, suggesting that K77, D80, E114, and E115 within C proteins were important for the inhibitory action.

Infection of the Cm3′ virus exhibited little IFN-β induction and dsRNA generation, although it has lost the ability to inhibit poly(I:C)-mediated translocation of IRF-3 into the nucleus, supporting above-mentioned relationship between viral abilities to generate dsRNA and to induce IFN-β, and suggesting that the viral dsRNA might be a major trigger of host innate immunity. In contrast, the Cm* virus has the ability to inhibit poly(I:C)-induced, MDA5-mediated translocation of IRF-3, although dsRNA production and significant induction of IFN-β was observed. This difference between these two viruses might implicate the difference of molecules triggering the innate immunity; viral RNA species, such as 5′-triphospated RNAs including leader RNA and non-encapsidated DI-genomes, other than dsRNA, might be major triggers of IFN-β induction in the Cm*-infected cells.

Of note, all recombinant viruses used in this study express V protein that has been reported to inhibit MDA5-mediated IFN-β production by its direct interaction with MDA5 [33], [34]. However, some of the recombinants, 4C(−), Cm2′, Cm3′, and D80A, failed to inhibit poly(I:C)-mediated translocation of IRF-3. This discrepancy might be due to the difference in the experimental system used, as mentioned above; cDNA and live recombinant virus systems. A previous study using genetically modified mice knocked out RIG-I and MDA5 genes has shown that infection of most of the RNA viruses including paramyxoviruses is recognized preferentially by RIG-I rather than by MDA5, despite functional interaction between their V proteins and MDA5 [27]. The V-MDA5 interaction might have a marginal effect on inhibition of virus-induced innate immunity. We are currently examining additional recombinant viruses containing mutations within the C as well as V proteins *in vitro* and *in vivo* to elucidate their functions in virus infection.

Finally, C proteins also have the ability to inhibit apoptosis induced by the infection. Lack of C protein and an F170S mutation within the C protein led to a quicker and severer CPE in the infected cells, although the WT-SeV infection had little CPE. Surprisingly, the Cm2′ and D80A viruses induced a much quicker and severer CPE associated with apoptosis than the 4C(−) virus, although all of the viral proteins of the D80A virus were the same as those of the WT virus except for a single D80A mutation within the C protein. It has been reported that the level of CPE induced by SeV infection is correlated with the level of IRF-3-stimulated arginase II [35]. Indeed, levels of CPE in the cells infected with the recombinant viruses are almost correlated with levels of IFN-β production in the infected cells. The inhibitory effect of C proteins on virus-induced apoptosis might be due to the combined effects of the C proteins to minimize production of viral RNA species sensed by RIG-I and MDA5 and to inhibit the signaling from recognition of viral RNA to activation of IRF-3, followed by IRF-3-stimulated gene expression including IFN-β and agrinase II, rather than due to the action of the C proteins to inhibit certain apoptotic pathways.

SeV C proteins exert multiple actions to finally produce infectious viral particles with maximum efficiency by utilizing and preventing cellular functions and regulating viral RNA synthesis. Here, we showed that C proteins play critical roles in viral escape from the host innate immunity and innate immune response-associated apoptosis. The C mutant viruses used in this study showed no remarkable difference from the wt virus in replication in cell cultures. However, most of the recombinant viruses had lost the ability to block IFN-α-triggered signaling leading to the establishment of an antiviral state in the infected cells. Infection of some of the recombinant viruses highly induced IFN-β production and apoptotic cell death. In addition, our results strongly suggested that C protein inhibited poly(I:C)-mediated activation of IRF-3 leading to IFN-β production as well as regulated viral RNA synthesis to avoid recognition of viral RNA by RIG-I and MDA5. Only single or double amino acid substitutions within C protein resulted in such a variety of viral phenotypes, implying that the abilities of the C protein observed above might be based on a common molecular mechanism. Since all of the recombinant viruses, except for C′/C(−) and 4C(−), showed virtually no growth defect in the cell cultures, we are currently examining the replication and pathogenicity of these viruses in mice, and *in vitro* experiments to elucidate the molecular mechanisms for multiple functions of C protein are underway. Further understanding of viral strategies for escape from host innate immunity and for maximizing production of infectious particles will hopefully lead to a better understanding of viral pathogenesis and the development of novel therapeutics to target important steps in viral replication.

## Materials and Methods

### Cells, viruses, and antibodies

LLC-MK_2_, CV1, BHK-21, Vero, and human HeLa cells were maintained in Dulbecco's minimum essential medium (DMEM; Nacalai Tesque, Kyoto, Japan) supplemented with 10% fetal bovine serum (FBS; Biological Industries, Kibbutz, Israel) and penicillin-streptomycin (Invitrogen) at 37°C. The polyclonal antibody (pAb) against the entire virion of SeV was described previously [36]. The pAbs against SeV P and C proteins were kindly provided by A. Kato (National Institute of Infectious Diseases, Japan). The monoclonal antibody (mAb) against SeV N protein was kindly provided by E. Suzuki (National Institute of Infectious Diseases, Japan). The pAb against green fluorescent protein (GFP) (sc-8334; Santa Cruz biotechnology, Santa Cruz, CA), pAb against human interferon regulatory factor 3 (IRF-3) (sc-9082; Santa Cruz biotechnology), mAb against double-stranded RNA (dsRNA) (J2; Scicons, Hungary), Alexa 488-conjugated anti-mouse IgG and Alexa 546-conjugated anti-rabbit IgG goat pAbs (Invitrogen), and horseradish peroxidase (HRP)-conjugated anti-rabbit IgG goat pAb (Santa Cruz Biotechnology) were used according to the protocols of the suppliers. Vaccinia virus expressing T7 RNA polymerase, vTF7.3 [37], was kindly provided by B. Moss (National Institutes of Health, USA), and propagated in CV1 cells. A recombinant VSV expressing GFP (rVSV-GFP) was kindly provided by K. Shinozaki (Hiroshima Prefectural Hospital, Japan) and propagated in BHK-21 cells. The SeV recombinants, C′/C(−) and 4C(−) [23] and C* [10], were kindly provided by A. Kato. All SeV recombinants and Newcastle disease virus (Herts strain) were propagated in embryonated chicken eggs. Titers of SeV recombinants were determined by an immunofluorescent infectious focus assay in LLC-MK_2_ cells and expressed as cell infectious units (CIU)/ml, as described previously [36].

### Construction and recovery of SeV recombinants

The Plasmid pSeV(+) encoding the full-length SeV cDNA (Z strain) was kindly provided by A. Kato. Mutations were introduced into the C ORF of the P gene using a QuickChange site-directed mutagenesis kit (Stratagene) to yield Cm2′, Cm3′, Cm4′, D80A, and F170S. None of these mutations resulted in alterations to the P polypeptide from an overlapping ORF. These P genes were inserted back into pSeV(+) to generate the full-length cDNA clones used to recover infectious viruses. SeV recombinants were recovered as described previously by Kato *et al.* [38] or by Nishimura *et al.* [39]. The mutations were confirmed by direct DNA sequencing of the RT-PCR products of viral genomic RNAs prepared from the purified virions.

### One-step growth curves of SeV recombinants

LLC-MK_2_ cells in six-well plates were infected with SeV mutants at a multiplicity of infection (MOI) of 5. After 1h at 37°C, inocula were removed, and cells were washed with phosphate-buffered saline (PBS) three times and incubated with serum-free DMEM containing 20 µg/ml of trypsin (Merck) at 37°C for 48 h. The culture medium was harvested at the indicated time points, clarified at 3,000 rpm for 10 min., and titrated in duplicate with LLC-MK_2_ cells.

### Virion protein profiles

LLC-MK_2_ cells in 10cm-diameter dishes were infected with SeV recombinants at an MOI of 5, as described above. At 48 h post-infection (p.i.), the culture medium was harvested and clarified at 3,000 rpm for 10 min. Virions were then centrifuged at 40,000 rpm for 2 h through a 20% sucrose cushion in a Beckman SW55 rotor. The pellet was suspended in SDS-PAGE sample buffer (125 mM Tris-HCl [pH 6.8], 4.6% SDS, 10% 2-mercaptoethanol, 0.005% bromophenol blue, and 20% glycerol) and analyzed by SDS-PAGE (8%). Gels were stained with SYPRO tangerine protein gel stain (Invitrogen), and analyzed using a FLA-3000G fluorescent image analyzer (Fuji Film). Cell lysates were also prepared and analyzed by Western blotting using pAbs against P and C proteins and a mAb against N protein. Protein bands were visualized using an Immobilon Western Chemiluminescent HRP substrate (Millipore), and analyzed using a chemiluminescence imaging system (LAS-1000plus, Fuji Film).

### RNA preparation

LLC-MK_2_ or HeLa cells in six-well plates were infected with SeV recombinants at an MOI of 5, as described above. At 24 or 48 h p.i., the culture medium was harvested and clarified at 3,000 rpm for 10 min. Samples were treated with 20 µg/ml of RNase A (Novagen) and 2 U of DNase I (Novagen) at 37°C for 1 h. Viral RNA was then prepared using a QIAamp viral RNA mini kit (QIAGEN). Cells were also harvested by trypsinization, and then total RNA was prepared using an RNeasy mini kit (QIAGEN).

### Quantitative RT-PCR

Quantitative RT-PCR was performed as described previously [18]. Briefly, for the detection of (−) and (+)-sense viral genomic RNAs, first strand cDNAs were synthesized using a QuantiTect Reverse Transcription kit (QIAGEN) with viron-derived RNA samples prepared above and either 5SeVZ1683 or 3SeVZ1843 as a primer to detect (−) or (+)-sense RNAs, respectively. The cDNAs were then applied to a quantitative real time PCR (qPCR) using a QuantiFast SYBR green PCR kit (QIAGEN) with the cDNAs prepared above as templates and the primer set 5SeVZ1683+3SeVZ1843, and analyzed using a DNA Engine Opticon 2 continuous fluorescence detection system (Bio-Rad). For the detection of IFN-β mRNA in the infected cells, RT-PCR was performed using a QuantiFast SYBR Green RT-PCR kit (QIAGEN) with the RNA samples prepared above from the infected cells as templates and the primer set 5hIFNb1-481-500+3hIFNb1-640-661 designed to amplify the region from nt 481 to 661 of the human IFN-β1 mRNA, and analyzed using DNA Engine Opticon 2. The amplification of specific DNA fragments during the reaction was confirmed by measuring the melting temperature of the PCR products and separating the products on an agarose gel.

### Cytotoxicity assay

LLC-MK_2_ cells in six-well plates were infected with SeV mutants at an MOI of 5, as described above. At 24 and 48 h p.i., cells were observed using a light microscope (Nikon ECLIPSE TE2000-S). At 48 h p.i., cytotoxicity in the infected samples was assayed using a CytoTox 96 Non-Radioactive Cytotoxicity Assay kit (Promega) according to the instructions supplied.

### Detection of double-stranded RNA

Vero cells cultured on glass coverslips or in six-well plates were infected with the indicated viruses at an MOI of 5, as described above. At 24 h p.i., cells were fixed with a 0.5% formaldehyde solution, permeabilized with 0.1% Triton X-100 and treated with 1% H_2_O_2_ in PBS(−). Cells were stained with an anti-dsRNA mAb as a primary antibody, and visualized using an HRP-conjugated goat anti-mouse IgG antibody as a secondary antibody and the Tyramide Signal Amplification Cyanine 3 System (Perkin Elmer). Coverslips were mounted on glass slides and observed using a Zeiss LSM 5 confocal microscope (Carl Zeiss).

### Poly (I:C) treatment and Immunofluorescence microscopy

HeLa cells cultured on glass coverslips were infected with SeV mutants at an MOI of 5, as described above. At 12 h p.i., the culture medium was replaced with fresh serum-free DMEM and 5 ug of poly (I:C) (GE Healthcare) was transfected using Lipofectamine 2000 (Invitrogen). After an additional 6 h at 37°C, cells were fixed with the 0.5% formaldehyde solution, and treated with 0.1% Triton X-100 in PBS. Cells were then stained using a mAb against SeV N and a pAb against human IRF-3, as primary antibodies, and Alexa 488-conjugated anti-mouse IgG and Alexa 546-conjugated anti-rabbit IgG goat pAbs as secondary antibodies. Coverslips were mounted on glass slides and observed using the confocal microscope.

### IFN-α treatment of rSeV-infected cells

HeLa cells in six-well plates were infected with the indicated viruses at an MOI of 5. After a 1-h incubation at 37°C, inocula were removed, and cells were washed with PBS three times and incubated with serum-free DMEM at 37°C. At 6 h p.i., the culture medium was replaced with fresh serum-free DMEM containing IFN-α (1,000 IU/ml; R&D Systems). After an additional 6-h incubation, the culture medium was removed, and cells were superinfected with rVSV-GFP at an MOI of 3. After 1 h at 37°C, inocula were removed, and cells were washed with PBS three times and further incubated with serum-free DMEM at 37°C for 6 h. GFP expression was observed using a fluorescence microscope (Nikon ECLIPSE TE2000-S). Cells were then lysed in SDS-PAGE sample buffer, and proteins were analyzed by SDS-PAGE (12%) followed by Western blotting using pAbs against GFP and SeV P and C proteins as primary antibodies, and HRP-conjugated anti-rabbit IgG goat pAb as a secondary antibody. Protein bands were visualized and analyzed as described above.
